# Prevalence and factors associated with transmission of schistosomiasis in school-aged children in South Sudan: a cross-sectional study

**DOI:** 10.11604/pamj.supp.2022.42.1.34006

**Published:** 2022-06-07

**Authors:** Mutale Nsakashalo Senkwe, Kibebu Kinfu Berta, Samuel Makoy Yibi, Julia Sube, Alex Bidali, Abias Abe, Adiele Onyeze, Jane Pita Hilary Ajo, John Rumunu Pascale, Fabian Ndenzako, Olushayo Oluseun Olu

**Affiliations:** 1 World Health Organization, Country Office, Juba, South Sudan,; 2 Ministry of Health, Juba, South Sudan,; 3 National Public Health Laboratory, Ministry of Health, Juba, South Sudan,; 4 Multicountry Assignment Team, World Health Organisation, Nairobi, Kenya

**Keywords:** Schistosomiasis, S. haematobium, S. mansoni, bilharzia, circulating cathodic antigen, Kato Katz, urine filtration, haematuria, mapping surveys, South Sudan

## Abstract

**Introduction:**

South Sudan is affected by a high burden of Neglected Tropical Diseases (NTDs). The country is very vulnerable to NTDs due to its favourable tropical climate and multiple risk factors. However, the distribution of the diseases and the populations at risk for the various NTDs is unknown. This paper described the distribution of schistosomiasis in 58 counties and 261 schools in South Sudan.

**Methods:**

a descriptive quantitative cross-sectional study of schistosomiasis in 58 counties in 8 states of South Sudan recruited school-aged children. Using different laboratory techniques, the children were tested for Schistosoma mansoni (*S. mansoni*) and Schistosoma haematobium (*S. haematobium*). A quantitative descriptive statistical was performed to determine the prevalence rates and the endemicity of schistosomiasis among 13,286 school-aged children.

**Results:**

the overall prevalence of *S. mansoni* and *S. haematobium* were 6.1% and 3.7% using Kato Katz and urine filtration concentration testing techniques. The highest state prevalence was reported in Western Equatoria for both *S. mansoni* (14.7%) and *S. haematobium* (7.3%). The age of the participants varied from 4 to 18 years; of these, children 10 to 12 years old had the highest prevalence of *S. mansoni* (6.8%) and *S. haematobium* (3.7%). The prevalence of *S. mansoni* (7% male vs 5% female) and *S. haematobium* (3.6% male vs 3.1% female) were higher in males than females. The likelihood of the prevalence of *S. mansoni* in males was 1.42 (95% CI:1.23, 1.64) higher than in females, while for *S. haematobium*, 1.36 (95% CI:1.12, 1.65) higher than in females. The prevalence of *S. mansoni* and *S. haematobium* showed a statistically significant gender difference (P< 0.05).

**Conclusion:**

the study had provided evidence of the distribution of schistosomiasis in South Sudan for policy direction and recommended annual preventive chemotherapy with praziquantel in all endemic areas.

## Introduction

Schistosomiasis, bilharzia, or snail fever is a common neglected tropical disease (NTD), highly endemic in the tropics and subtropical areas [1]. It is caused by trematode parasites of the genus Schistosoma which has five species known to infect and cause morbidity in humans, namely *S. haematobium*, S. intercalatum, S. japonicum, S. mekongi, and *S. mansoni* [2]. Sexual reproduction occurs in the human host, where fertilized eggs are produced and released through urine and stool. The eggs trapped in tissues induce inflammation and die [3]. In freshwater, free-living miracidia released by the eggs infect the snail and undergo asexual replication to release the cercariae, which penetrates the skin of humans who come into contact with the infested water [2]. The African region is predominantly affected by *S. mansoni* and *S. haematobium* due to the availability of freshwater where the intermediate hosts thrive well. S. japonicum and S. mekongi are confined to Asia, while the Caribbean and Latin America are affected mainly by *S. mansoni* [4]. Schistosomiasis is the commonest NTD affecting at least 230 million people globally. Sub-Saharan Africa contributes 90% of the disease burden [1-3].

Acute schistosomiasis, or “Katayama Syndrome” is a short-lived hypersensitivity reaction related to tissue migration of the larva. Acute infection presents as fever, myalgia, headache, cough, and rash. The symptoms appear 14-84 days after infection and may last for several weeks [5]. At the same time, chronic diseases include bladder cancer, blood in the stool, constipation, diarrhoea, bowel wall ulceration, fibrosis, hyperplasia, polyposis, and portal hypertension [5,6].

In South Sudan, hospital records have indicated ongoing schistosomiasis transmission in Upper Nile, Jonglei, Eastern and Central Equatoria states with two species, *S. mansoni* and *S. haematobium*. The eastern and central parts of South Sudan including Jongile, Unity, Upper Nile state, and Greater Pibor Administrative Area (GPAA) is prone to frequent flooding. Abnormally heavy rainfall causes the overflow of Lol, Nile, Pibor, Sobat and other rivers that lead to inland flooding where people reside and causes displacement. In addition, the flooding increases the chance of contact between people and the parasite carrying an intermediate host [7,8]. A survey conducted in 2009 in Northern Bahr el Ghazal showed that *S. haematobium* is endemic mainly in areas along the Lol River [9]. In a 2010 survey, both *S. mansoni* and S.haematobium were endemic throughout Unity and some foci in Central Equatoria and Eastern Equatoria States [10]. These observations suggested that schistosomiasis was present, and transmission was ongoing; however, the country’s actual geographical distribution, extent, and prevalence remain unknown. South Sudan is yet to scale up and sustain control activities, ensure access to essential medicines, and complement public health interventions in response to the resolution of World Health Assembly 54 (WHA54) schistosomiasis and soil-transmitted helminth infections [11]. To adequately implement control and elimination measures, and monitor and assess the impact of interventions, accurate estimates of the disease burden and prevalence are crucial. The manuscript describes the national distribution of human schistosomiasis in 58 counties from 2016 through 2019.

## Methods

**Study design and area:** we conducted a cross-sectional quantitative study to know the prevalence of schistosomiasis among school-aged children 10 to 14 years of age in South Sudan from 2016 to 2019. The study was based on the methods and procedures stated recommendation under WHO neglected tropical diseases transmission assessment survey guidelines [12].

**Study site selection and sample size:** South Sudan is administratively divided into 10 states which are further divided into 79 counties. Twenty-two counties in four states, with available recent schistosomiasis prevalence data, were identified and excluded from the current study. The states without prevalence and transmission risk data were included in the study. Besides, the selection was guided by previous knowledge of ecological factors (i.e., proximity to lakes, streams, and other water bodies) from different geographical locations [13]. It had helped the management of survey implementation including logistics constraints. All 52 counties in the six remaining states and six counties (Panyijar county from Unity and Kapoeta South, Kapoeta North, Torit, Magwi, and Lapon counties from Eastern Equatoria) were included in the study with a total of 58 counties. In these counties, the selection of schools was based on the WHO survey sample builder stated in the assessment guideline [12]. It is an excel based tool used to develop survey design and random samples. It had facilitated the random selection of schools and children from a list of randomized numbers. A cluster-based simple random sampling technique was applied to select five schools in each county. Before the section of the schools, all schools in the county were listed down for random sections. Fifty children aged between 10 and 14 years, balanced by gender, from each school were randomly enrolled on the study. WHO NTD transmission assessment survey guideline recommends 50 children per cluster [12]. When the target was not reached, children from an expanded age range were selected. Otherwise, the target age from the surrounding villages or the nearest school was sampled to complete the required samples. Each child submitted a stool and urine sample to test the presence of intestinal Schistosoma (*S. mansoni*) eggs and urinary Schistosoma (*S. haematobium*), respectively.

**Data collection:** the data collection was conducted by nine teams, each comprising a supervisor (i.e. laboratory technologist or an experienced laboratory technician), two laboratory technicians, a data clerk and a social mobilizer. A data manager coordinated the work of all data clerks and had access to the Open Data Kit (ODK) web-based cloud to monitor data as entries were made and uploaded using smartphones. A detailed fieldwork plan was developed based on the teams created and the counties included in the study. The local health personnel led the study teams to the selected schools. Geographical coordinates (i.e. longitude, latitude, and altitude) were taken at the survey sites using smartphones. Children aged between 10 to 14 years were randomly selected, and both urine and stool samples were collected from the selected children. The survey determined the prevalence of *S. haematobium* based on micro-hematuria or parasite eggs in urine using reagent dipstick and urine filtration procedures, respectively. A reagent dipstick for hematuria is a qualitative rapid diagnostic tool used to screen school-aged children for urinary schistosomiasis. The interpretation of the result was based on the presence or absence of a test strip line. Samples with red test lines were labelled as positive; however, further quantification of the test line was not performed. The urine filtration procedures determine the concentration of the egg per 10 millilitres (ml) of the urine sample. The testing result was reported as light: <50 ova per 10ml; heavy: ≥50 ova per 10ml.

The prevalence of *S. mansoni* was determined parasite eggs in stool using Kato Katz or circulating cathodic antigen (CCA) in urine. The Kato Katz technique is used for semi-quantitative and quantitative diagnosis of Schistosoma species (*S. mansoni*), soil-transmitted helminthiasis caused by Trichuris trichiura, hookworm, and ascaris lumbricoides. WHO had recommended the use of the technique in areas with moderate to high transmission of intestinal schistosomiasis (10-49.9% and ≥50%) [14]. A gram of faeces was collected and tested for the presence of an egg (egg per gram of faeces) using a microscope. The testing result was reported as light infection (0-99 e.p.g.), medium (100-399 e.p.g.), and heavy: (≥400 e.p.g.). A point-of-care Circulating Cathodic Antigen (POC-CCA) urine test is a simplified rapid diagnostic test (RDT) used for the qualitative detection of an active *S. mansoni* infection. The test detects Schistosoma specific antigen execrated in urine [15]. The result was labelled positive based on the appearance of a visible test line seen within 20 minutes of application of the urine. The prevalence estimate for each survey site was classified into non-endemic (0%), low (<10%), moderate (10-49.9%), or high-risk (≥50%). The prevalence estimate for each survey site was classified into non-endemic (0%), low (<10%), moderate (10-49.9%), or high-risk (≥50%) areas.

**Data analyses:** in 2016, data were captured on to a server hosted at the national level using Bold Like Us (BLU) Studio 5.5 smartphones running Android 4.2 (Jelly Bean) through a modified version of Open Data Kit (ODK), the LINKS application. Raw data on schistosoma infection were downloaded into Microsoft Excel, cleaned, and summarized from the server. In subsequent years, the Expanded Special Project for Elimination of Neglected Tropical Diseases (ESPEN) Collect application was used to capture, facilitate cleaning, and aggregation collected individual data into site data. The data entered was synchronized and hosted at the Standard Code Server. Only authorized officers had access to download individual data from the dashboard on the server. We conducted descriptive analyses on socio-demographic characteristics and epidemiological distribution of schistosomiasis. We have used International Business Machine (IBM) Statistical Package for Social Science Window Version 26.0 (SPSS V26) to conduct descriptive epidemiology. A prevalence map was produced using ArcGIS (ESRI, California, and the USA). A two-by-two table was used to determine the relationship between test types (urine filtration versus dipstick and CCA versus Kato Katz) and test result (positive versus negative). A two-by-two table was performed to compare whether the observed prevalence of *S. haematobium* and *S. mansoni* results for the two test types for each species were statistically different enough to reject the null hypothesis (there is no significant difference between the two test types) at a 95% confidence interval (CI) and a significant level of 0.05.

**Ethical consideration:** during the 2016, 2018, 2019 survey ethical approval was obtained from the Research and Ethics Committee of the national Ministry of Health. During the survey, consent was obtained from the headteachers and parents. All positive cases found during the study received a single dose of 20 mg/kg praziquantel. However, ethical approval was not obtained to use the secondary anonymized data in the analysis. Data protection measures were employed to ensure the security of the data.

## Results

**Study population and schistosoma infection prevalence:** the screening of the 13,286 samples collected from the children revealed the presence of both *S. haematobium* (n=441) and *S. mansoni* (n=804) Table 1. The age of the children tested varied from 4 to 18 years old. The age group 10 to 12 years had the highest prevalence rate for *S. mansoni, S. haematobium*, and Co-infection of the two species. Of the 6,982 males tested for *S. mansoni*, 7% tested positive. The prevalence of *S. mansoni* in males was 1.42 (95% CI: 1.23,1.64) times higher than in females. Regarding *S. haematobium*, 3.8% of males were tested positive compared to 2.8% positive females. The prevalence of *S. haematobium* in males was 1.36 (95% CI:1.12,1.65) times greater than in females.

**Table 1:** socio-demographic characteristics of schistosomiasis in school-aged children, South Sudan (2016 to 2019)

Variables	Category	Number tested (N)	*S. mansoni*		*S. haematobium*		Co-infection	
			Tested positive	Prevalence (%)	Tested positive	Prevalence (%)	Tested positive	Prevalence (%)
State	Eastern Equatoria	1130	23	2	3	0.3	0	0
	Jonglei	2833	331	11.7	172	6.1	102	3.6
	Lakes	1822	94	5.2	3	0.2	0	0
	Unity	99	2	2	17	17.2	0	0
	Upper Nile	2764	11	0.4	92	3.3	7	0.3
	Warrap	1606	4	0.2	31	1.9	0	0
	Western Bar el Ghazal	745	2	0.3	2	0.3	2	0.3
	Western Equatoria	2287	337	14.7	167	7.3	114	5
Gender	Male	6982	488	7.0	248	3.6	125	1.9
	Female	6304	316	5.0	193	3.1	110	1.6
Age group	0-4	6	0	0.0	0	0/0	0	0.0
	5-9	1085	72	6.6	19	1.8	16	1.5
	10-12	7232	494	6.8	267	3.7	210	2.9
	13-15	4958	238	4.8	155	3.1	9	0.2
	>15	5	0	0.0	0	0.0	0	0.0

Note: the prevalence of S. mansoni and S. hamatobium was calculated using the stool Kato-Katz testing and urine filtration concentration respectively. The two tests were conducted in 2016, 2018 and 2019; however, circulating cathodic antigen (CCA)-(S. mansoni) and urine dipstick was only available in 2016.

**Figure 1:**
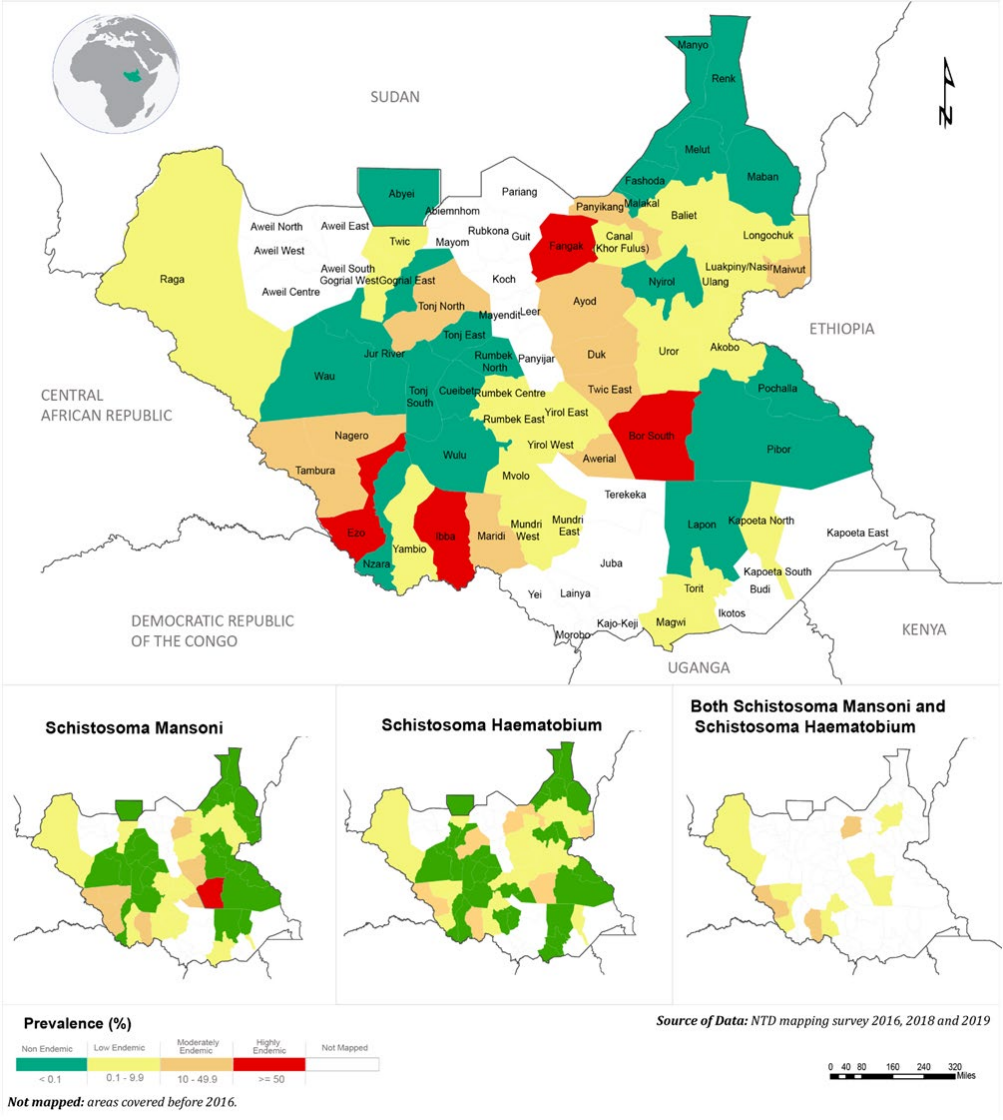
schistosomiasis endemicity map, South Sudan (2016 to 2019)

**Prevalence of schistosoma infection by location:** *S. mansoni* infection was found in all the eight states of South Sudan, with an overall prevalence rate of 6.1% as shown in Table 2. Western Equatoria had the highest prevalence rate of S. manosni (14.7%). The prevalence rate varied from 0 to 65.9% at the county level, with the highest in Bor South (65.9%). At the school level, 86% out of 261 schools were endemic to *S. mansoni*. The school prevalence rate of S. manosni varied from 0 to 93.3%; the highest was recorded at Bandala primary school in Nagero county (93.3%) of Western Equatoria state. *S. haematobium*, infection was found in all eight states with an overall prevalence rate of 3.7%. Unity state had the highest prevalence rate (17.2%) Table 2. At the primary school level, 72 (%) out of 261 schools were endemic to *S. haematobium*. The school prevalence rate varied from 0% to 84.4%, with the highest at Toch primary school in Old Fangak (84.4%) in Jonglei state. Overall, high endemicity for schistosomiasis combined is in Bor South, Old Fangak, Ezo and Ibba counties as indicated in Figure 1. Bor South county has highest the endemicity for *S. mansoni* species.

**Table 2:** summary of Schistosoma mansoni laboratory test, South Sudan (2016 to 2019)

State	Stool: Kato-Katz (*S. mansoni*)					Urine: circulating cathodic antigen (CCA)- (*S. mansoni*)	
	Number of stools tested	Prevalence rate (%)	Infection intensity (eggs per gram of faeces) [% of positive)			Number of samples tested	Prevalence rate (%)
			Light	Medium	Heavy		
Eastern Equatoria	1130	2.0	1.9	0.1	0.0	1130	15.6
Jonglei	2833	11.7	9.5	1.3	0.9	840	34.0
Lakes	1822	5.2	4.9	0.1	0.1	1822	12.8
Unity	99	2.0	2.0	0.0	0.0	-	-
Upper Nile	2764	0.4	0.4	0.0	0.0	-	
Warrap	1606	0.2	0.2	0.1	0.0	1406	18.1
Western Bar el Ghazal	745	0.3	0.3	0.0	0.0	-	
Western Equatoria	2287	14.7	13.6	1.1	0.3	649	30.8
Total	13286	6.1	5.3	0.5	0.3	5847	19.7

Intensity by Kato-Katz: light: 0-99 egg per gram of faeces (e.p.g.); medium: 100-399 e.p.g.; heavy: ≥ 400 e.p.g; intensity by urine filtration: light: < 50 ova per 10 ml; heavy: ≥ 50 ova per 10 ml; point of care-Schistosoma circulating cathodic antigen (POC-CCA) was used in 2016 in five states.

**Prevalence of parasitic intensity:** light intensity infection (1-99 egg per gram of faeces (e.p.g.) for *S. mansoni* by Kato Katz technique was observed in all the states that varied from 0.2 and 13.6% as indicated in Table 3. Western Equatoria recording the highest. Medium intensity infections (100-399 e.p.g. of faeces) were observed in five out of the eight states at 1.3% in Jonglei, 1.1% in Western Equatoria and 0.1% each in Eastern Equatoria, Lakes and Warrap states and heavy intensity infection (>400 e.p.g. of faeces) was observed in three states of Jonglei at 0.9%, Western Equatoria 0.3% and Lakes 0.1%. For *S. haematobium* using urine filtration, light intensity infection (<50 eggs/10mls of urine) was observed in the eight states with the highest in Unity (16.2%), Western Equatoria (4.4%) and Jonglei (3.8%), while heavy intensity infections (>50 eggs/10mls of urine) were observed in all except Western Bahr el Ghazal.

**Table 3:** summary of Schistosoma haematobium laboratory test, South Sudan (2016 to 2019)

State	Urine: filtration concentration				Urine dipstick	
	Number of samples tested	Prevalence (%)]	Infection intensity (eggs per 10 ml) [% of positives]		Number of samples collected	Prevalence (%)
			Light	Heavy		
Eastern Equatoria	1130	0.3	0.1	0.2	-	-
Jonglei	2833	6.1	3.8	2.3	1993	3.2
Lakes	1822	0.2	0.1	0.1	-	-
Unity	99	17.2	16.2	1.0	99	26.3
Upper Nile	2764	3.3	2.3	1.0	2764	3.6
Warrap	1606	1.9	0.7	1.2	200	0.0
Western Bar el Ghazal	745	0.3	0.3	0.0	745	0.3
Western Equatoria	2287	7.3	4.4	2.9	1638	3.9
Total	13286	3.7	2.3	1.4	7439	3.4

Note: Intensity by urine filtration: light: < 50 ova per 10 ml; heavy: ≥ 50 ova per 10 ml

Overall, the *S. mansoni* prevalence rate for 2016 surveys using Circulating Cathodic Antigen (CCA) was 19.7%, while for the Kato Katz technique was 7.8%. The likelihood of the test being positive was 2.87 (95% CI: 2.54, 3.24) higher for CCA than Kato Katz. There was a statistically significant difference between the two tests (P<.001) (Table 4). As for *S. haematobium*, the overall prevalence rate using urine filtration was 5.0%, while for dipstick was 3.4%. The likelihood of the test being positive was 1.49 (95% CI: 1.27, 1.75) higher in urine filtration compared to the dipstick. There was a statistically significant difference between the two tests (P<.001) (Table 4).

**Table 4:** comparison of the performance of different tests used for *S. mansoni* and *S. haematobium* testing, South Sudan (2016 to 2019)

Test type	Positive	Negative	Total	Odds ratio	P-value	95% confidence interval
circulating cathodic antigen	1149(19.7%)	4698(80.3%)	5847 (100%)			
Kato-Katz	400(7.8%)	4698 (92.8)	5098 (100%)	2.87	<.001	(2.54, 3.24)
Total	1549(14.2%)	9396 (85.8)	10945 (100%)			
Urine filtration	375(5.0%)	7064(95.0%)	7439(100%)	1.49	<.001	(1.27, 1.75)
Urine dipstick	255(3.4%)	7184(96.6%)	7339(100%)			
Total	630(4.2%)	14248(95.8)	14878(100)			

## Discussion

The cross-sectional study provides the baseline data on schistosomiasis prevalence as the first sizeable school-based survey in the 58 unmapped counties of South Sudan. Our study revealed the presence of schistosomiasis infections in all the states and almost in every county with a heterogeneous prevalence rate of *S. haematobium* and *S. mansoni* across the country, indicating the latter to be highly endemic [16]. There is a high prevalence of *S. mansoni* in Jonglei, Western Equatoria and the Lakes States and *S. haematobium* in Jonglei, Unity and Western Equatoria States. Higher co-infection has been observed in Jonglei State. The parasitic intensity was light across the eight states, with heavy intensities observed more for *S. haematobium* than *S. mansoni*. Jonglei and the Western Equatoria States had co-infection of the two Schistosoma parasites [16]. Our findings showed the schistosomiasis burden in the central and southwestern regions of the country is high. The persistence of schistosomiasis’s high prevalence rate over the years indicates that no control or elimination measures are being implemented [16]. In Africa, the prevalence of human schistosomiasis is dependent on the level of environmental sanitation, the suitability of the area for the intermediate snail hosts, and the type of the snails. In South Sudan, this has been exacerbated by political conflict negatively impacting the country’s socio-economic status and funding for preventing and controlling infectious diseases such as schistosomiasis [17]. Previous surveys of 2010 showed a constantly lower prevalence of *S. haematobium* than that of *S. mansoni*, a similar finding in six of the eight states of our study a common feature in Madagascar due to the Biomphalaria snails in the Nile River and high positivity of *S. mansoni* [18]. One unexpected finding was the low prevalence of schistosomiasis in Eastern Equatoria (2.3%) compared to the previous survey findings of 14.2% 6 years prior. The conflicts in the region and mainly children (62% of children became refugees) and women fleeing insecurity, violence, and famine to Uganda, may elucidate the reduction [19,20]. Nonetheless, the rapid decline in prevalence rate requires further research to verify these findings and assess the contributing factors at length.

Previous schistosomiasis predictive maps for South Sudan suggested a high prevalence rate of *S. haematobium* in Greater Bahr El Ghazel between 10-25% and the Equatoria States at ≤5%, and findings of a non-endemic Western Bahr El Ghazal and low prevalence rate of 0.5% in Western Equatoria [21]. While the surveys of 2009 found a prevalence rate of 3% in Northern Bahr El Ghazal [9]. Deganello in 2007 reported the presence of *S. mansoni* mainly in Central Equatoria, with a national prevalence of both species between 10-25% again. This is inconsistent with our observed findings which are relatively lower with an overall prevalence rate of 7.9% (7.3-8.3% at 95% confidence interval) [22]. However, the conclusions of Jonglei were consistent with the predicted low prevalence of <5%. Overall, the current state-level data indicates an uneven distribution throughout the country, with a few previously thought-to-be highly endemic places found to be lowly or non-endemic. The prevalence of schistosomiasis from the initial prevalence rate surveys and our study is not surprising as no sustainable control measures were instituted.

An alarmingly (very high) prevalence rate of schistosomiasis was observed in Bor South, where schools recorded over 90% site prevalence rates. This high prevalence could be attributed to the long-running civil war that has devastated social and health services in the area and the presence of the schistosoma, intermediate host, and freshwater bodies. The high prevalence was seen in Bor South, Ibba, Ezo, Awerial, Nagero, and Old Fangak were comparable to other focal prevalence rate surveys in East African countries, Madagascar, Egypt, and the Democratic Republic of Congo, an indication of the high burden of schistosomiasis in South Sudan and many parts of the Africa Region [21,22]. Also, the observed high prevalence in rural areas compared to urban areas such as Wau may be attributed to inadequate sanitary facilities, contaminated water sources for domestic chores, bathing, and insufficient health education on the affected communities´ preventive measures. In some countries, urban infection is found in individuals due to urbanisation. This could be the assumption for the situation seen in Juba, the capital city of South Sudan in the state of Central Equatoria, where the prevalence is high [10]. Typically, if the intermediate host is not present in an area with schistosomiasis cases, it is easier to control through better chemotherapy, sanitation, and water access facilities [23].

Presently, chemotherapy is the most cost-effective approach to controlling schistosomiasis in the short term, but complimenting it with access to clean water and sanitation would help address the risk factors, reduce transmission, and have a broader impact on health. While the provision of water, adequate sanitation, and snail control are essential for schistosomiasis elimination, directing these interventions to selected high transmission areas would ensure a higher impact [4]. The study´s five sites per county is an innovative way to improve resource allocation for interventions to focal transmission. However, this is also a study limitation as some counties are enormous, and the five sites may not be represented precisely. The utilisation of WASH facilities through behavioural modification has been challenging in some countries despite being the best approach to managing health problems [3]. The communities require sufficient knowledge and attitude about schistosomiasis through health education in the endemic areas.

Preventive chemotherapy either school or community-based approaches have not shown significant differences in some countries following treatment [24]. Most children are exposed to similar conditions when they get home with inadequate WASH facilities. Therefore, most schistosomiasis treatment should be both community and school-based, with many children in communities. Treatment immediately after 12 months is vital because delayed repeat therapy may not suppress transmission due to persistent reinfections leading to reduced drug efficacy observed after one to three years [24]. Therefore, the state government should emphasise the need for additional non-drug control measures in highly endemic areas.

## Conclusion

This study shows that schistosomiasis is endemic in South Sudan, with a moderate to high prevalence rate in most parts of the country. In these endemic communities we advise the national schistosomiasis control programme to implement the WHO recommendation of annual preventive chemotherapy with a single dose of praziquantel at 75% treatment coverage in all the age groups from 2 years. The treatment should include pregnant women in the second and third trimester, lactating women and adults. In the low endemic communities, the test and treat approach is recommended. The low parasitic intensity for both species in South Sudan is an indication that with suitable interventions, the prevalence of infection in the affected population can reduce to elimination levels. The study has generated crucial epidemiological baseline information and indicators which could guide policy formulation, monitoring, and evaluation of interventions. Heightened health education to reduce contact with contaminated water and access to safe water, sanitation, and hygiene plus environmental interventions including snail control with moluscicides should be complementary measures to reduce the prevalence and burden of schistosomiasis. The effective implementation and monitoring of these strategies will interrupt transmission and eliminate schistosomiasis in South Sudan. The verification for the interruption of transmission should use diagnostics with high sensitivity and specificity.

### What is known about this topic

- Although schistosomiasis is endemic in South Sudan, the geographic distribution of the disease is unclear;
- The superior sensitivity of circulating cathodic antigen over KK in the detection of the S. mansoni circulating cathodic antigen.

### What this study adds

- This study provides information on the prevalence and distribution of schistosoma infection in South Sudan;
- The younger children are at higher risk of S. mansoni infection;
- Key recommendations for scaling up effective and integrated public health measures for prevention and control of schistosomiasis including treatment of the pre-school age children.
- Malaria prevention practices are sub-optimal compared to the national targets.

## References

[1] Gabrielli AF, Montresor A, Chitsulo L, Engels D, Savioli L (2011). Preventive chemotherapy in human helminthiasis: theoretical and operational aspects. Trans R Soc Trop Med Hyg.

[2] World Health Organization (2013). Schistosomiasis: progress report 2001 - 2011, strategic plan 2012 - 2020.

[3] Colley DG, Bustinduy AL, Secor WE, King CH (2014). Human schistosomiasis. Lancet Lond Engl.

[4] Lu XT, Gu QY, Limpanont Y, Song LG, Wu ZD, Okanurak K (2018). Snail-borne parasitic diseases: an update on global epidemiological distribution, transmission interruption and control methods. Infect Dis Poverty.

[5] Engels D, Zhou XN (2020). Neglected tropical diseases: an effective global response to local poverty-related disease priorities. Infect Dis Poverty.

[6] Nelwan ML (2019). Schistosomiasis: life cycle, diagnosis, and control. Curr Ther Res Clin Exp.

[7] Guo SY, Li L, Zhang LJ, Li YL, Li SZ, Xu J (2021). From the one health perspective: schistosomiasis japonica and flooding. Pathogens.

[8] Wu XH, Zhang SQ, Xu XJ, Huang YX, Steinmann P, Utzinger J (2008). Effect of floods on the transmission of schistosomiasis in the Yangtze River valley, People´s Republic of China. Parasitol Int.

[9] Sturrock HJW, Picon D, Sabasio A, Oguttu D, Robinson E, Lado M (2009). Integrated mapping of neglected tropical diseases: epidemiological findings and control implications for Northern Bahr-el-Ghazal State, Southern Sudan. PLoS Negl Trop Dis.

[10] Finn TP, Stewart BT, Reid HL, Petty N, Sabasio A, Oguttu D (2012). Integrated rapid mapping of neglected tropical diseases in three states of South Sudan: survey findings and treatment needs. PLoS One.

[11] World Health Assembly (1997). Elimination of lymphatic filariasis as a public health problem.

[12] World Health Organization Control of neglected tropical diseases.

[13] Rebollo MP, Onyeze AN, Tiendrebeogo A, Senkwe MN, Impouma B, Ogoussan K (2021). Baseline mapping of neglected tropical diseases in Africa: the accelerated WHO/AFRO mapping project. Am J Trop Med Hyg.

[14] Bharti B, Bharti S, Khurana S (2018). Worm infestation: diagnosis, treatment and prevention. Indian J Pediatr.

[15] Casacuberta-Partal M, Hoekstra PT, Kornelis D, van Lieshout L, van Dam GJ (2019). An innovative and user-friendly scoring system for standardised quantitative interpretation of the urine-based point-of-care strip test (POC-CCA) for the diagnosis of intestinal schistosomiasis: a proof-of-concept study. Acta Trop.

[16] Rumunu J, Brooker S, Hopkins A, Chane F, Emerson P, Kolaczinski J (2009). Southern Sudan: an opportunity for NTD control and elimination?. Trends Parasitol.

[17] (2018). ISPI Environmental vulnerability: South Sudan´s endgame.

[18] Spencer SA, Penney JMSJ, Russell HJ, Howe AP, Linder C, Rakotomampianina ALD (2017). High burden of Schistosoma mansoni infection in school-aged children in Marolambo District, Madagascar. Parasit Vectors.

[19] United Nations Security Council Working group on children and armed conflict.

[20] UN HRC (2017). Report of the commission on human rights in South Sudan (A/HRC/34/63) (advance edited version). ReliefWeb, South Sudan.

[21] Secor WE, Wiegand RE, Montgomery SP, Karanja DMS, Odiere MR (2020). Comparison of school-based and community-wide mass drug administration for schistosomiasis control in an area of western kenya with high initial Schistosoma mansoni infection prevalence: a cluster randomized trial. Am J Trop Med Hyg.

[22] Woodall PA, Kramer MR (2018). Schistosomiasis and infertility in East Africa. Am J Trop Med Hyg.

[23] Silva LK, Barbosa LM, Kovach JD, Teixeira RDS, Soares ES, Cardoso CW (2020). The changing profile of schistosomiasis in a changing urban landscape. Int J Parasitol.

[24] Onkanga IO, Mwinzi PNM, Muchiri G, Andiego K, Omedo M, Karanja DMS (2016). Impact of two rounds of praziquantel mass drug administration on Schistosoma mansoni infection prevalence and intensity: a comparison between community-wide treatment and school-based treatment in western Kenya. Int J Parasitol.

